# Molecular Mechanisms of Action of Genistein in Cancer: Recent Advances

**DOI:** 10.3389/fphar.2019.01336

**Published:** 2019-12-06

**Authors:** Hardeep Singh Tuli, Muobarak Jaber Tuorkey, Falak Thakral, Katrin Sak, Manoj Kumar, Anil Kumar Sharma, Uttam Sharma, Aklank Jain, Vaishali Aggarwal, Anupam Bishayee

**Affiliations:** ^1^Department of Biotechnology, Maharishi Markandeshwar (Deemed to be University), Mullana-Ambala, India; ^2^Division of Physiology, Zoology Department, Faculty of Science, Damanhour University, Damanhour, Egypt; ^3^NGO Praeventio, Tartu, Estonia; ^4^Department of Chemistry, Maharishi Markandeshwar University, Sadopur, India; ^5^Department of Animal Sciences, Central University of Punjab, Bathinda, India; ^6^Department of Histopathology, Post Graduate Institute of Medical Education and Research, Chandigarh, India; ^7^Lake Erie College of Osteopathic Medicine, Bradenton, FL, United States

**Keywords:** genistein, cancer, proliferation, apoptosis, metastasis, *in vitro*, *in vivo*

## Abstract

**Background:** Genistein is one among the several other known isoflavones that is found in different soybeans and soy products. The chemical name of genistein is 4′,5,7-trihydroxyisoflavone. Genistein has drawn attention of scientific community because of its potential beneficial effects on human grave diseases, such as cancer. Mechanistic insight of genistein reveals its potential for apoptotic induction, cell cycle arrest, as well as antiangiogenic, antimetastatic, and anti-inflammatory effects.

**Objective:** The purpose of this review is to unravel and analyze various molecular mechanisms of genistein in diverse cancer models.

**Data sources:** English language literature was searched using various databases, such as PubMed, ScienceDirect, EBOSCOhost, Scopus, Web of Science, and Cochrane Library. Key words used in various combinations included genistein, cancer, anticancer, molecular mechanisms prevention, treatment, *in vivo*, *in vitro*, and clinical studies.

**Study selection:** Study selection was carried out strictly in accordance with the statement of Preferred Reporting Items for Systematic Reviews and Meta-analyses.

**Data extraction:** Four authors independently carried out the extraction of articles.

**Data synthesis:** One hundred one papers were found suitable for use in this review.

**Conclusion:** This review covers various molecular interactions of genistein with various cellular targets in cancer models. It will help the scientific community understand genistein and cancer biology and will provoke them to design novel therapeutic strategies.

## Introduction

Genistein [5,7-dihyroxy-3-(-4-hydroxyphenyl)-4H-1-benzopyran-4-one] is a major isoflavone in soy and soy-based food products that are regularly consumed by people in Asian countries (Ronis, 2016). Indeed, median daily intake of isoflavones among adults in Japan and China is about 25–50 mg which is several-folds higher than the consumption of these compounds by women in the western countries (less than 3 mg) (Sak, 2017a). Several epidemiological studies have suggested a lower incidence of certain cancer types, such as breast and prostate cancer, in Asian countries as compared to the western world. Therefore, recent interest has been focused on the possible contribution of high dietary consumption of isoflavones for prevention and suppression of tumorigenesis (Sak, 2017b). In the past few decades, numerous studies have been published about the potential anticancer role of genistein both in cell cultures as well as animal models (Spagnuolo et al., 2015; Kim et al., 2014). This natural isoflavone can exhibit a wide range of important properties, including antioxidant, anti-inflammatory, antiangiogenic, proapoptotic, and antiproliferative activities, all of which confer chemopreventive, and chemotherapeutic potential of genistein (Kim et al., 2014; Ganai and Farooqi, 2015; Uifalean et al., 2015).

Despite extensive studies, cancer has remained one of the gravest diseases and biggest challenges for human health all over the world, being a leading cause of death in the industrialized countries. For more than half a century, various chemotherapeutic drugs have been developed and used for treatment of tumors; however, there are still no curative options currently available in clinical settings (Crawford, 2013). Therefore, natural agents with strong anticancer activities could be considered as novel potential lead compounds for the development of more efficient and selective anti-neoplastic drugs (Bishayee, 2012; Shanmugam et al., 2016). Genistein may be one of such lead compounds; it is safe and its anticancer efficiency has already been proven in numerous preclinical investigations. This soy isoflavone can affect various molecular targets and modulate various signaling pathways, thereby influencing the ultimate response of cancer cells (Spagnuolo et al., 2015; Russo et al., 2016; Ardito et al., 2018; Chae et al., 2019). However, besides the anticancer effects, contradictory action has also been described for genistein by promoting growth of malignant cells (Russo et al., 2016), making studies with this molecule a scientific challenge. Therefore, in this article, molecular mechanisms of genistein in malignant cells are unraveled and analyzed.

## Methodology for Literature Search

Various authentic and reliable databases, such as PubMed, EBOSCOhost, ScienceDirect, Scopus, Web of Science, and Cochrane Library, were used to search and collect literature. The Preferred Reporting Items for Systematic Reviews and Meta-Analysis (PRISMA) criteria (Liberati et al., 2009) has been followed. Relevant full-length articles published in peer-reviewed journals until June, 2019 were included. Conference abstracts, book chapters, and unpublished results were not included. Only English language articles were included in this review. Major keywords used in various combinations included: genistein, cancer, anticancer, molecular mechanisms prevention, treatment, *in vivo*, *in vitro*, and clinical studies. The bibliography of the primary literature was also studied to collect additional relevant articles.

## Chemistry of Genistein

**Figure 1:**
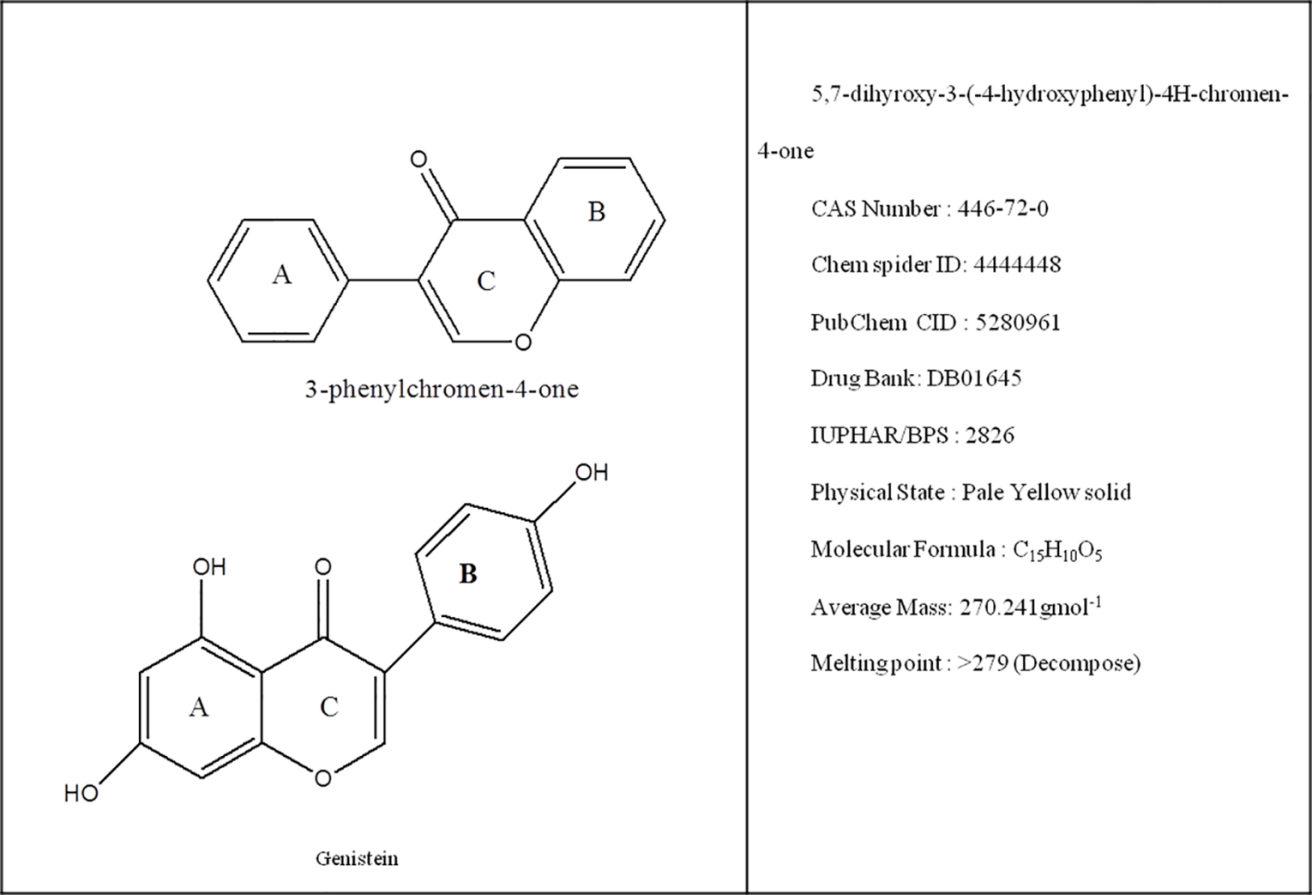
Representation of basic nucleus of isoflavones along with chemical and physical properties of genistein.

Genistein belongs to isoflavone family and is obtained from soy products, such as soybeans. It is very well-known plant secondary metabolite that consists of the 3-phenylchromen-4-one nucleus made up of two aromatic rings (A and B) (**Figure 1**). Further, these rings are linked to another carbon pyran ring (C) (**Figure 1**). Other functional group in its basic carbon skeleton are C_2_–C_3_ double bond, an oxo group at C4 position of ring C. Additionally, there are three hydroxyl groups at C5, C7, and C4′ positions of ring A and B, respectively (**Figure 1**). Genistein was first isolated in 1899 from a species of flowering plant *Genista tinctoria* (Dyer’s broom) of family Fabaceae (Perkin and Newbury, 1899). Following the initial discovery, it has been found in many plants, such as lupin, fava beans, soybeans, and kudzu. Genistein was successfully synthesized first time in 1928 (Baker and Robinson, 1928).

**Figure 2:**
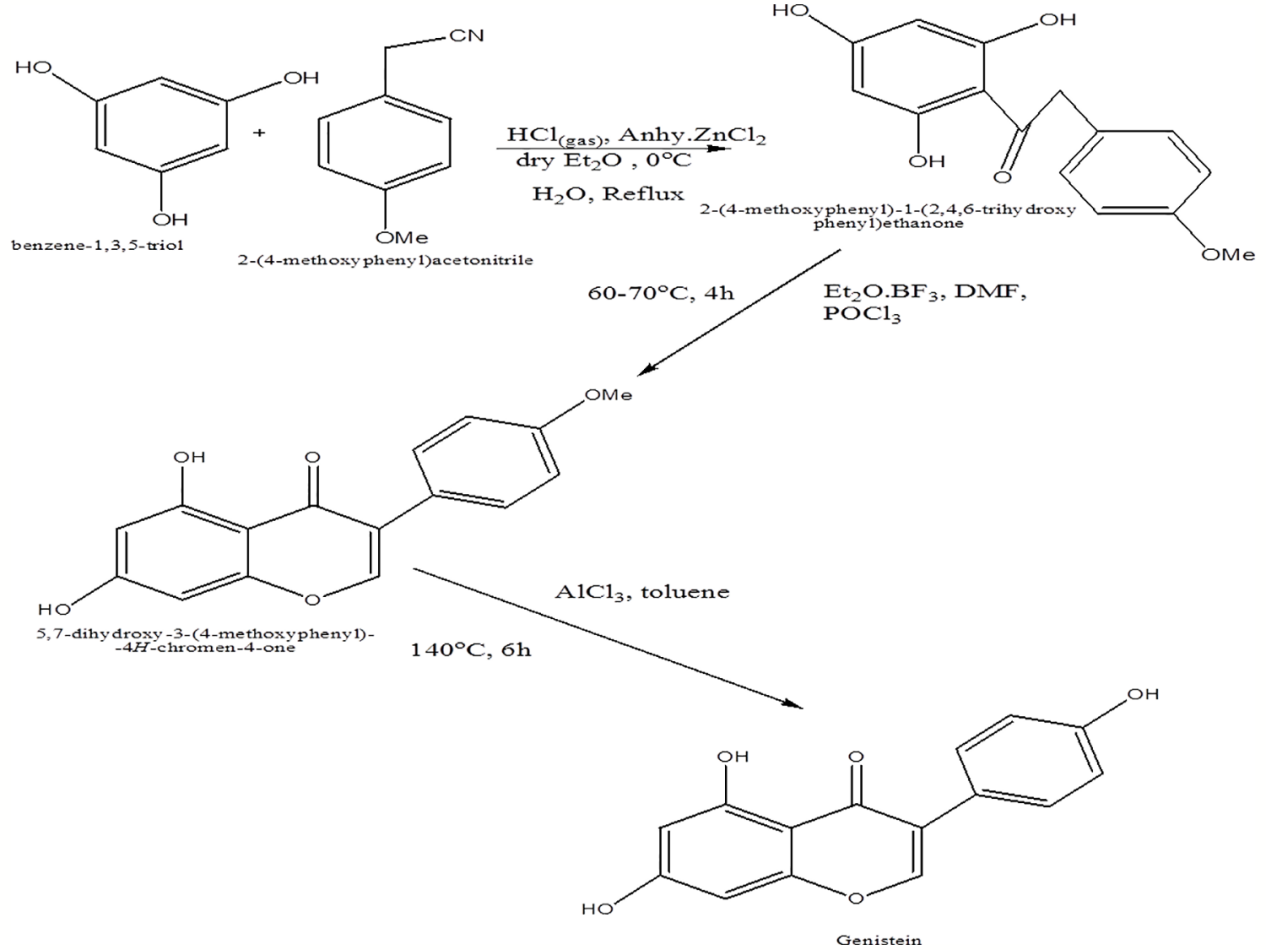
Synthesis route of genistein *via* trihydroxybenzoin.

**Figure 3:**
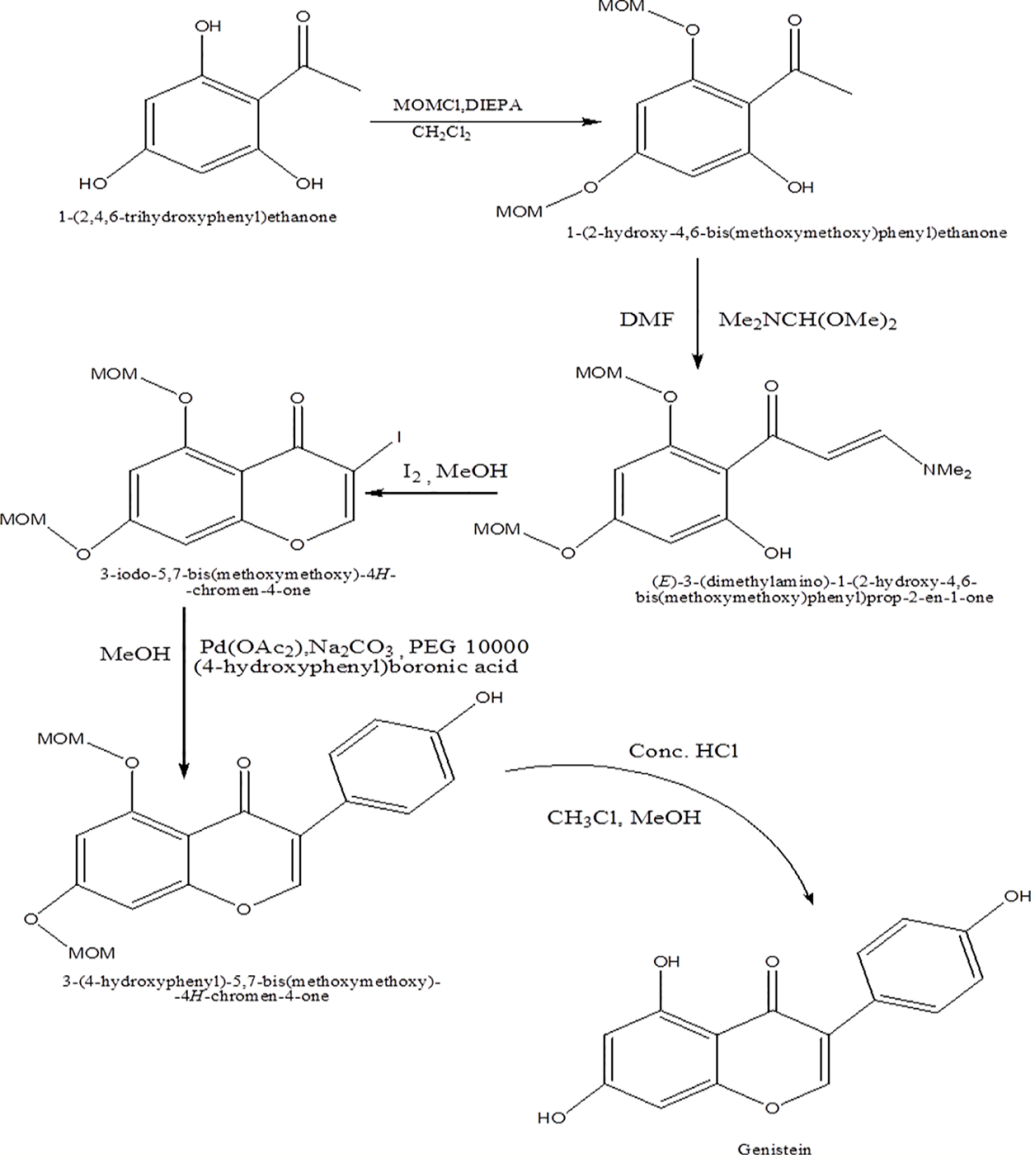
Synthesis of genistein from 1-(2,4,6-trihydroxyphenyl)ethanone.

Genistein is synthesized by treatment of trihydroxybenzoin, which, in turn, is obtained *via* acylation of phloroglucinol, with substituted phenyl acetonitrile using HCl and anhydrous ZnCl_2_ in dry ether as catalyst (**Figure 2**) (Hamza Sherif and Gebreyohannes, 2018). In another approach, genistein has been synthesized from 2,4,6-trihydroxyphenyl) ethanone *via* protection of two hydroxyl substituent in triol as methoxymethyl ester so as to overcome the problem of reaction of dimethoxy methyl dimethylamine with phenol (**Figure 3**) (Denis et al., 2010).

The derivatives of genistein can also be achieved by Ferrier rearrangements of 3,4-di-*O*-acetyl-L-rhamnal with 3-bromopropanol to 2,3-unsaturated bromoalkylglycosides followed by epoxidation with meta-chloroperoxybenzoic acid and subsequently connected with genistein (Goj et al., 2012). Additionally, genistein derivatives are also derived through novel chemical glycosylation and glycoconjugation process (Szeja et al., 2017).

## Molecular Mechanisms of Action of Genistein

The molecular mechanism of action of genistein as a chemotherapeutic agent has been extensively studied in different types of cancers. Genistein modulates various steps of cell cycle, apoptosis, angiogenesis, and metastasis. The main molecular targets of genistein include caspases, B-cell lymphoma 2 (Bcl-2), Bcl-2-associated X protein (Bax), nuclear factor-κB (NF-κB), inhibitor of NF-κB, phosphoinositide 3-kinase/Akt (PI3K/Akt), extracellular signal-regulated kinase 1/2 (ERK 1/2), mitogen-activated protein kinase (MAPK), and Wingless and integration 1/β-catenin (Wnt/β-catenin) signaling pathway. Besides the transcription factors, genistein-induced endoplasmic reticulum (ER) stress and its downstream targets are also reported to induce apoptosis in cancer. Interestingly, peroxisome proliferator-activated receptors (PPARs) have also surfaced as potential therapeutic targets of interest for modulating tumor growth, and genistein has been documented to induce apoptosis in tumor cells *via* targeting PPARγ signaling cascade. The underlying molecular mechanism of action of genistein in apoptosis induction, cell cycle arrest, anti-inflammatory potential along with inhibition of angiogenesis, and metastasis are discussed in detail in the following sections and summarized in **Table 1**.

**Table 1:** Anticancer molecular mechanism of genistein.

Effect	Mechanism	Cancer model	Reference
Apoptosis-evasion	ER-stress and mitochondrial involvement, ↑ATF-6α, ↑GRP-78, ↑Bax, ↑Bad, ↑Bak	HL-60	Hsiao et al. (2019)
	↓PKL1		Chae et al. (2019
	↓MMP, ↑ROS	Mia-PaCa2 and PANC-1	Bi et al. (2018)
	↑Capase-3	HT29	Shafiee et al. (2016)
	↓MDM2, ↓XIAP, ↓caspase-3	HeLa	Xie et al. (2013)
	↓CIP2A mRNA with modulation of E2F1	MCF-7-C3 and T47D	Zhao et al. (2016)
	TRAIL-mediated apoptotic cell death,↑LC3-II,↑p62	A549	Nazim et al. (2015)
Cell cycle arrest	G_2_/M arrest	HL-60	Hsiao et al. (2019)
	G_0_/G_1_arrest by cell cycle transition	MCF-7/ERβ1	Jiang et al. (2018)
	G_0_/G_1_arreast	Mia-PaCa2 and PANC-1	Bi et al. (2018)
	Mitotic arrest, ↓PlK1	TP53-mutated A460 cancer cells	Shin et al. (2017)
	G_2_/M arrest	HCT116	Mizushina et al. (2013)
	G_2_/M arrest by telomere shortening *via* suppression of TR and TERT mRNA	Glioblastoma multiforme and medulloblastoma cells	Khaw et al. (2012)
Antimetastastic	↓MMP2	HT29	Shafiee et al. (2016)
	↓DMBA-induced metastatic transition	Mouse model	Banerjee et al. (2008)
Anti-inflammatory	Decreases TNF-α-induced fractalkine expression	THP-1	Sung et al. (2010)
Antiproliferative	↑p-ERK, ↑pCREB, ↑BDNF, ↓AChE	Mouse model	Lu et al. (2018)
	↓mTOR, ↓p70S6K1, ↓4E-BP1, ↓NF-kB, ↓Bcl-2, ↑Nrf2, ↑HO-1, ↑Bax	Hen model	Sahin et al. (2019)
	↓DNMTs, ↓HDACs	HeLa cells	Sundaram et al. (2018)
	↓DNA methylation, ↑ATM, ↑APC, ↑PTEN, ↑SERPINB5	MCF-7 and MDA-MB-231	Xie et al. (2014)
	↓Crosstalk between ERα and IGF-IR pathway, ↑BPA ↑estrogen	BG-1	Hwang et al. (2013)
	↓Topoisomerase II	HCT116	Mizushina et al. (2013)
	↑ERα expression, ↑TAM-dependent antiestrogen therapeutic sensitivity	ERα-positive MCF-7 and ERα-negative MDA-MB-231 and MDA-MB-157 cells	Li et al. (2013)
	↓MMP-2, ↓VEGF	Glioma cells	Yazdani et al. (2016)
	↑p53,DKK1, ↓HDAC4/5/7, ↓DVL, ↓BAX, ↓survivin, ↓phospho MEK	SK-UT-1, MES-SA-Dx5, MES-SA	Yeh et al. (2015)

### Induction of Apoptosis

**Figure 4:**
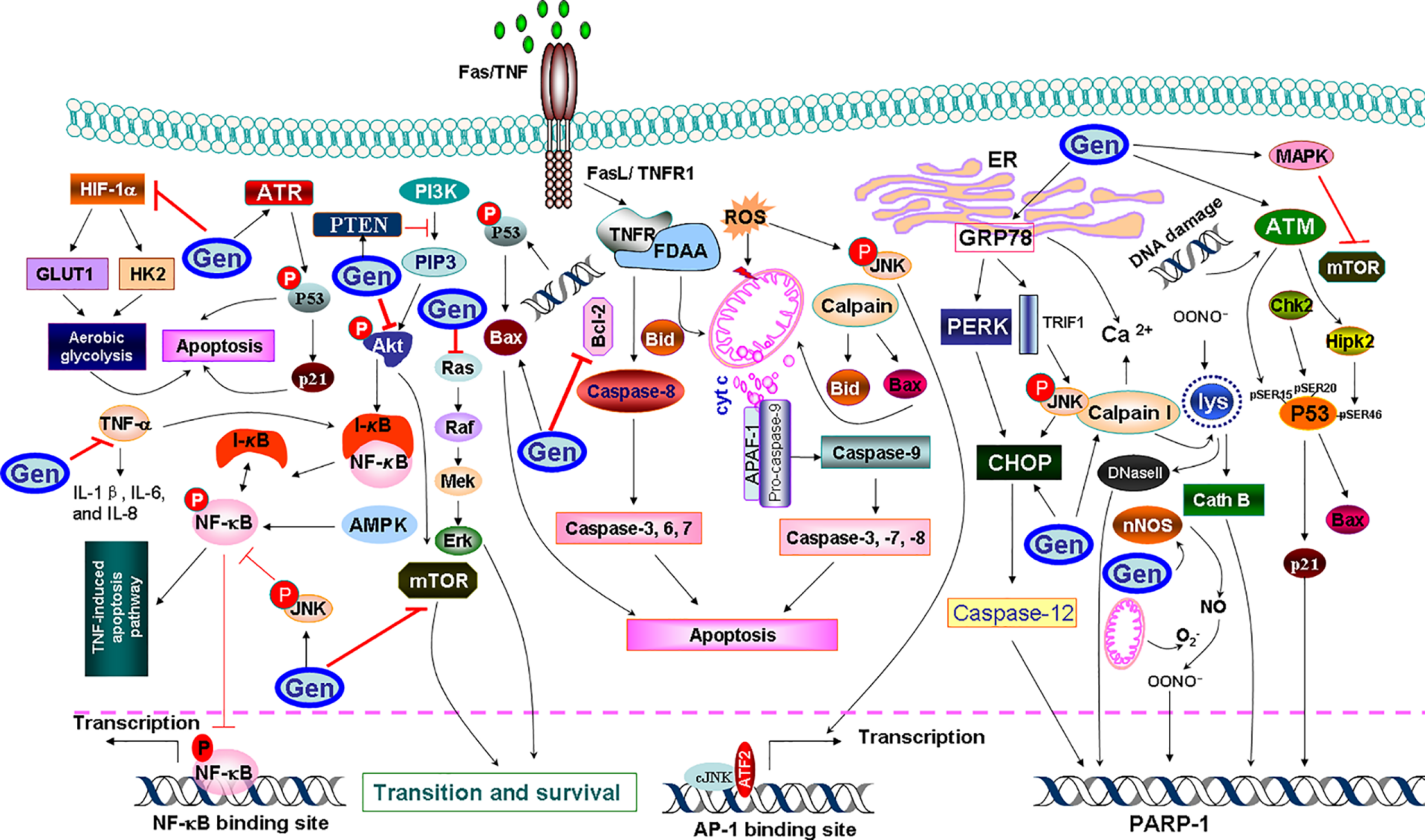
A diagram of mechanistic insight of genistein (Gen) to induce apoptosis in cancer cells. Genistein increases the endoplasmic reticulum (ER) stress-associated protein expressions, such as calpain 1, which cleaves cytosolic Bax and Bid that truncated AIF (tAIF) and cytochrome *c* (cyt *c*) release, along with the activation of the apoptotic protease activator factor 1 (APAF1). Genistein triggers the ER stress through upregulation of glucose-regulated protein 78 (GRP78) expression, which increases the activity of protein kinase R-like ER kinase (PERK), which, in turn activates the transcription factor CCAAT/enhancer-binding protein homologous protein (CHOP) to induce apoptosis. To mediate the extrinsic apoptosis, genistein triggers the combination of a ligand (FasL/TNFR1) and a death receptor (Fas/TNF). Meanwhile, genistein triggers the combination of a ligand (FasL/TNFR1) and a death receptor (Fas/TNF) to mediate the extrinsic pathway. Genistein abolishes tumor necrosis factor-α (TNF-α)-induced nuclear factor-κB (NF-κB) translocation as well as phosphorylation of IκB kinase-α/β and IκBα. Genistein also enhances the phosphorylation and activation of ATM/ATR-p53-p21 signaling pathway. Calpain I also permeabilizes lysosomal (lys) membranes, resulting in release of cathepsin B (Cath B) and DNase II. Genistein downregulates hypoxia-inducible factor-1α (HIF-1α), therefore inactivating glucose transporter 1 (GLUT1) or/and hexokinase 2 (HK2), which in turn suppresses aerobic glycolysis and mediates apoptosis.

Apoptosis, a programmed cell death, is mainly characterized by a series of distinct changes in cell morphology, such as blebbing, loss of cell attachment, cytoplasmic contraction, DNA fragmentation, and other biochemical changes, including the activation of caspases through extrinsic and/or intrinsic mitochondrial pathways. Past research has demonstrated the role of genistein in the induction of apoptosis through regulation of expression of various proteins (**Figure 4**). Genistein was able to induce apoptosis in human cervical cancer cells (HeLa cells) by enhancing the activities of each of caspase-9 and caspase-3, and/or both (Dhandayuthapani et al., 2013). Additionally, genistein could trigger apoptotic cell death by inhibiting NF-κB pathway and modulating the levels of antiapoptotic protein Bcl-2 and proapoptotic protein (Bax) in LoVo and HT-29 colon cancer cell lines (Luo et al., 2014). Genistein also promoted apoptosis in HT29 colon cancer cells by modulating caspase-3 and p38 MAPK signaling pathway (Shafiee et al., 2016).

In another context, genistein-mediated apoptosis *via* induction of ER stress was recently proposed as a potential mechanism for the induction of apoptosis in human cervical cancer cells *via* upregulating the expression of glucose-regulated protein 78 (GRP78) and CCAAT/enhancer-binding protein homologous protein (Yang et al., 2016). Recently, a study using Hepa1-6 hepatocellular carcinoma cells has also confirmed the concentration-dependent apoptotic effect of genistein (Sanaei et al., 2018). In this regard, the release of cytochrome c (cyt c) and activation of the apoptotic protease activator factor 1 from the mitochondria are the marginal factors in the induction of apoptosis. One of the possible mechanisms is that genistein induced the activity of the Ca^2+^-dependent enzyme, namely calpain, which cleaves cytosolic Bax and Bid which, in turn, truncated apoptosis-inducing factor and cyt c release (Das et al., 2010). The other possible mechanism is that genistein increased the expression of ER stress-associated proteins, such as inositol-requiring enzyme 1α, calpain 1, 78 kDa GRP78, growth arrest, and DNA damage-inducible gene 153, caspase-7, and caspase-4. The upregulation of full form of GRP78 was involved in the ER stress (Hsiao et al., 2019). The induction of the latter upregulated the activity of protein kinase R-like ER kinase, which, in turn, activates the transcription factor CCAAT/enhancer-binding protein homologous protein to induce apoptosis (Xia et al., 2019). It has also been postulated that genistein enhanced the phosphorylation and activation of p53, while decreased the ratio of Bcl-2/Bax and Bcl-xL/Bax and the level of phosphorylated Akt (also known as protein kinase B), which result in cells undergoing apoptosis (Ouyang et al., 2009). Genistein-mediated activated protein kinase (AMPK) upregulation increased apoptosis of hepatocytes, in hepatocellular carcinoma (HCC) through energy-dependent caspase pathway (Lee et al., 2019). In Hep3B cells, genistein activated AMPK, which promoted apoptosis accompanied by downregulation of the proinflammatory responses and increasing mitochondrial respiration. Genistein has a potent anti-inflammatory effect by decreasing the secretion of interleukin-1β (IL-1β), IL-6, and IL-8 from tumor necrosis factor-α (TNF-α)-stimulated MH7A cells. Furthermore, genistein prevented TNF-α-induced NF-κB translocation into nucleus as well as phosphorylation of inhibitor of κBα (IκBα) and IκB kinase-α/β, and also suppressed TNF-α-induced AMPK inhibition (Li et al., 2014), which halted cellular proliferation and their survival. Another possible mechanism for the ability of genistein to mediate apoptosis came from its ability to induce phosphorylation of the ataxia-telangiectasia mutated (ATM) protein family and ATM-related (ATR) and activation of Chk1 and Chk2 checkpoint kinases (Ouyang et al., 2009), which in turn, phosphorylates and inactivates of phosphatases Cdc25C and Cdc25A. The sequence of the latter event includes inactivation of Cdc2, causing cell arrest in G2/M phase, and mediating apoptosis.

On the other context, genistein can trigger oxidative stress-induced apoptosis *via* increasing nitric oxide (NO) production and its bioavailability. In support of this hypothesis, it has been reported that genistein enhanced the activity of neuronal nitric oxide synthases (Miao et al., 2018), which promoted NO synthesis from l-arginine substrate. The latter can interact with superoxide (O^2−^) to form peroxynitrite (OONO^−^) and hydroxyl radical (OH^−^) that triggers poly(ADP-ribose) polymerase 1 activation, which initiates apoptosis. Lastly but not the least, genistein could mediate apoptosis through inhibiting aerobic glycolysis through downregulation of hypoxia-inducible factor-1α which inactivates glucose transporter 1 or/and hexokinase 2 (Li et al., 2017).

Further, genistein has also been reported to induce apoptosis via PPARγ pathway which involves PPARγ, Bcl-2, phosphatase and tensin homolog (PTEN), p21^Waf11/Cip1^, survivin, and cyclin B1. It has been reported that administration of genistein in combination with three polyunsaturated fatty acids, namely docosahexaenoic acid, eicosapentaenoic acid, and arachidonic acid, increased PPARγ expression in MDA-MB-231 human breast cancer cells, and decreased expression of inflammatory molecules cyclooxygenase-2 (COX-2) and prostaglandin E_2_ (PGE_2_) thereby reverting invasiveness in breast cancer cells (Horia and Watkins, 2007). The growth-inhibitory effect of genistein has also been reported in MG-63 osteosarcoma (OS) cells treated with GW9662, an antagonist of PPARγ (Song et al., 2015). Genistein treatment of OS cells increased PPARγ expression and led to tumor cell apoptosis as a nontoxic activator of PPARγ. On the contradictory, genistein in combination with resveratrol was found to downregulate the expression of adipocyte specific proteins, CCAAT/enhancer binding protein-α and PPARγ, thereby inhibiting adipogenesis in 3T3-L1 cells which led to induction of apoptosis and promotion of lipolysis in 3T3-L1 adipocytes (Rayalam et al., 2007). This was further supported by another study that documented combination of genistein and daidzein induced downregulation of perilipin-1, Tip-47, and adipose differentiation-related protein family proteins leading to inhibition of lipid droplet accumulation and induction of apoptosis in HT-29 colon cancer cells (Liang et al., 2018). In view of the limited scientific evidence supporting regulation of PPAR via genistein, further studies are needed to decipher the precise mechanism of action of genistein via PPAR signaling pathway.

Thus, in view of outlined scientific literature, genistein has been extensively documented to induced cancer cell apoptosis via different signaling cascades from activation of caspases to induction of ER stress, oxidative stress, MAPK, and PPARγ pathway. The in vitro and in vivo results are illustrated to show promising role of genistein in inducing apoptosis in different cancer types. However, further research is still needed to pinpoint the precise intracellular target for maximizing efficacy of genistein as a therapeutic drug.

### Cell Cycle Arrest

**Figure 5:**
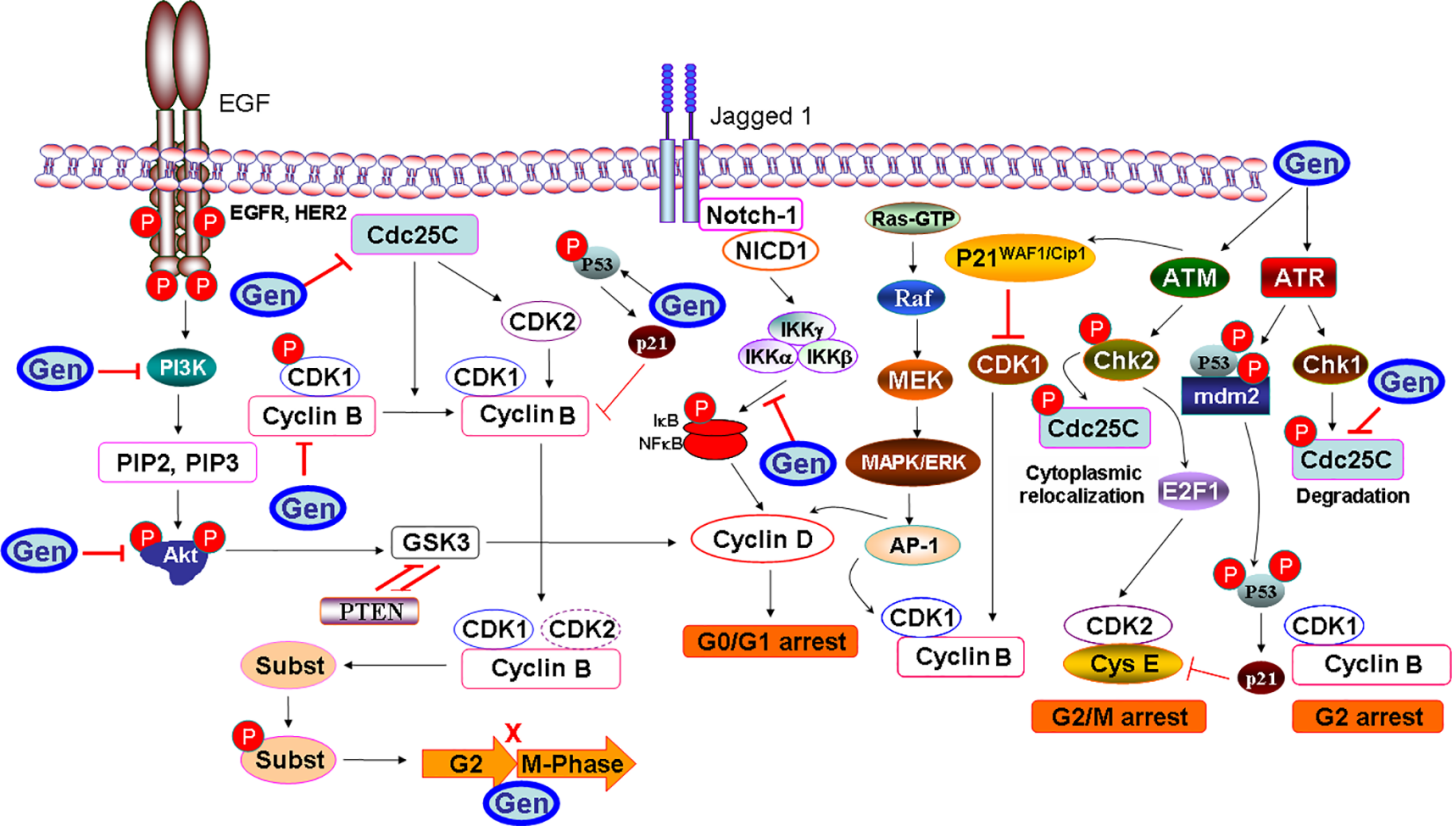
Molecular targets of genistein (Gen) in cancer cell cycle. Although genistein activates the expression of ATM protein family and ATM-related (ATR), and further activates the checkpoint kinases Chk2 and Chk1, which in turn phosphorylate Cdc25C. However, genistein promotes cytoplasmic relocalization and or degradation of Cdc25C, which would prevent cyclin-dependent kinase 1 (Cdk1)/cyclin B from being dephosphorylated and cause G2 arrest. ATM may also modulate transcriptional regulation of cell cycle progression through modification the expression of p53 and Mdm2, or through activation of Chk2, which can further modulate p53, E2F1 expression, leading to an upregulation in the p21 expression that would inhibit Cdk2 activity. ATM also phosphorylates the p53 tumor suppressor though the induction of Cdk inhibitor p21^Waf1/Cip1^. Genistein also could inactivate phosphatidylinositol-3-kinase (PI3K)/serine/threonine kinase (Akt)/glycogen synthase kinase 3 (GSK3) signaling pathway, whether through the prevention of the phosphorylation of Act and upregulation of the prime time entertainment network (PTEN) expression. Genistein may also mediate cell cycle arrest by suppressing NF-κB-mediated activation of cyclin D1 expression.

The cell cycle is the series of events that take place in a cell, leading to its division and duplication to produce two daughter cells. Genistein regulates the cell growth and cell cycle progression by modulating the expression of cell cycle-regulatory proteins (**Figure 5**). For instance, genistein inhibited the growth of human gastric carcinoma (HGC-27) cells by arresting the cell cycle succession at G_2_-M (Matsukawa et al., 1993). Similarly, in galectin-3-transfected human breast epithelial (BT549) cell line, it has been reported that genistein mediated G_2_/M cell cycle arrest via upregulation of the expression of p21^Waf11/Cip1^ (Lin et al., 2000). Using genistein-treated nonneoplastic human mammary epithelial (MCF-10F) cells, Frey et al. (2001) reported the downregulation of Cdc25C and upregulation of p21^WAF/CIP1^ expression. Similarly, genistein-treated neuroblastoma (B35) cells were found to show higher expression of Cdk inhibitor p21^Waf/Cip1^ (Ismail et al., 2006). Studies have also shown that genistein can trigger G_2_/M cell cycle arrest in human breast cancer (MDA-MB-231) cells via Ras/MAPK/activator protein-1 and consequently downregulating Cdk1, cyclin B1, and Cdc25C (Li et al., 2008). Further, it has been shown that genistein activated Chk1 and Chk2, which consequently inactivated Cdc25C and Cdc25A, Cdc2, resulting in G_2_/M phase cell cycle arrest (Ouyang et al., 2009). In addition, genistein can induce G_2_/M cell cycle arrest and apoptosis via ATM/p53-dependent pathway in human colon cancer (HCT-116 and SW-480) cells. Genistein was found to cause the cell cycle arrest in the G_2_/M phase via ATM/p53 and p21^Waf1/Cip1^. Additionally, the expression of Cdc2 and Cdc25A were also downregulated following genistein treatment (Zhang et al., 2013).

Such desired antiproliferative effect was also observed in normal human dermal fibroblasts, which would prevent scar formation in damaged and burned skin (Chin et al., 2000). This stringent ability of genistein to mediate cell cycle arrest in cancer cells would pose a question that does such cell cycle arrest is restricted to cancer and tumor tissue or could it be expanded to other non-malignant cells; or by other words, is the effect of genistein differ in other abnormal cases. In order to provide an answer for this question, it is very important to consider that although almost all cancer cells have mutations, other nonmalignant cells in the human body may carry mutations too. *For instance* mucopolysaccharidosis (MPS) type II fibroblasts are involved in inherited metabolic diseases caused by mutations that afflict the function of one of the degradation enzymes of glycosaminoglycans. The accumulation of such compounds in cells altered their proliferation and survival, ultimately leading to massive cellular dysfunctions. In fibroblasts derived from patients suffering from MPS types I, II, IIIA, and IIIB, genistein partially corrected the disturbances in the MPS II cell cycle and increased the tendency of MPS IIIA and IIIB fibroblasts to enter the S and G_2_/M phases (Moskot et al., 2016). Collectively, these data revealed that genistein not only confer a protection against the proliferation of cancer cells, but it could also interact with other pathological cases in a different manner.

### Antiangiogenesis Effects

**Figure 6:**
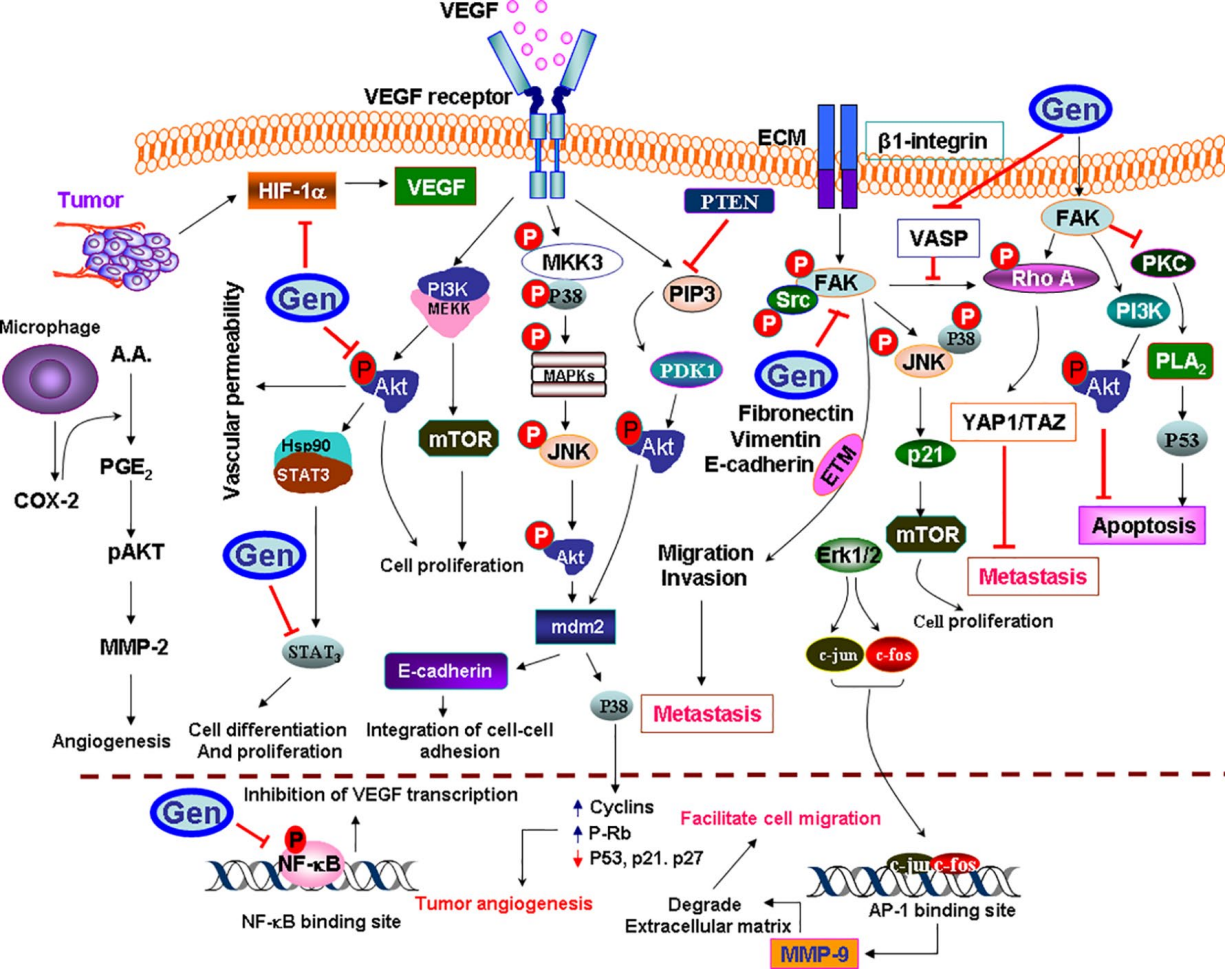
Schematic representation of antiangiogenic activity of genistein (Gen) in cancer. Genistein inactivates each of mitogen-activated protein kinases (MAPK), activator protein-1 (AP-1), Akt/NF-κB, and Erk1/2 pathways, which abolished the downstream matrix metalloproteinase-9 (MMP-9) synthesis at the transcription level that declines the proteolytic activity and impairs the cellular migration. Although focal adhesion kinase (FAK) would prevent apoptosis through inactivation of the cytosolic phospholipase A2 (PLA2) and p53, but it would mediate apoptosis through the activation of PI3K/Act pathway. Genistein inhibits the activation of proangiogenic proteins (ERK/Akt/mTOR). Genistein could prevent cell differentiation and proliferation by increasing the activity of the tumor suppressor PTEN, which blocks Akt activation though PI3K dephosphorylation, and also dephosphorylate PIP3 into phosphatidylinositol 4,5-bisphosphate (PIP2). Genistein also prevents metastasis and enforces cancer cells to colonization in their site by phosphoprotein (VASP) upregulation. Since, VASP activates the extracellular matrix (ECM)-mediated β1-integrin-FAK-RhoA–YAP1-TAZ signaling to enhance YAP1/TAZ protein abundance. Genistein inactivates the epithelial–mesenchymal-transition (EMT) proteins, which promotes metastasis, including the proangiogenesis factors HIF-1α and vascular endothelial growth factor (VEGF), all are upregulated by hypoxia. Genistein downregulates PI3K/Akt/HIF-1α and NF-κB signaling pathways. Cycloxygenase-2 (COX-2) regulates the production of *prostaglandin E_2_* *(PGE_2_)* from arachidonic acid (AA). PGE_2_-pAkt axis is involved in regulation of MMP-2 activity, which, in turn, induces angiogenesis.

Angiogenesis is characterized by the creation of new vessels from an existing vasculature, which being considered an important target in cancer treatment strategy. Since angiogenesis support the growth of cancer cells with vital factors, such as oxygen and nutrients, the inhibition of vasculature can be a pivotal strategy for cancer therapy. Previous studies have shown that genistein exhibited potent antiangiogenic properties. For example genistein-treated E6 and five human bladder cancer cell lines have exhibited anti-angiogenic effect *via* downregulating vascular endothelial growth factor (VEGF), platelet-derived growth factor, urokinase plasminogen activator, matrix metalloprotease-2 (MMP-2) and MMP-9 expression. Furthermore, natural vasculature inhibitors, such as plasminogen activator inhibitor-1, endostatin, angiostatin, and thrombospondin-1 were found to be activated (Su et al., 2005). In another study, it has been proposed that genistein-mediated suppression of VEGF *via* downregulation of c-Jun N-terminal kinase and p38, PTK/MAPK, and MMPs production (Yu et al., 2012). In another study, oral squamous cell carcinoma cells also demonstrated the downregulation of VEGF, basic fibroblast growth factor, and MMP-2 due to genistein treatment (Myoung et al., 2003). In addition, *in vitro* invasion assay and gelatin zymography using HSC-3 cells have also shown a decreased expression of VEGF. More recently, using thyroid cancer cells, a group of investigators revealed the downregulation of VEGF-A expression along with human telomerase reverse transcriptase, PTEN, NF-κB, and p21 (Ozturk et al., 2018). All these antiangiogenic molecular mechanistic insights have been summarized in **Figure 6**. These results as mentioned above indicate that targeting VEGF may represent an effective key factor to deter the subsequent and deleterious effect of activation of its downstream molecular pathways. By blocking it, whether by specific inhibitors or by genistein, we can downregulate a panel of molecular pathways that aid cancer cells to establish *de novo* formation of blood vessels.

### Antimetastasis Effect

**Figure 7:**
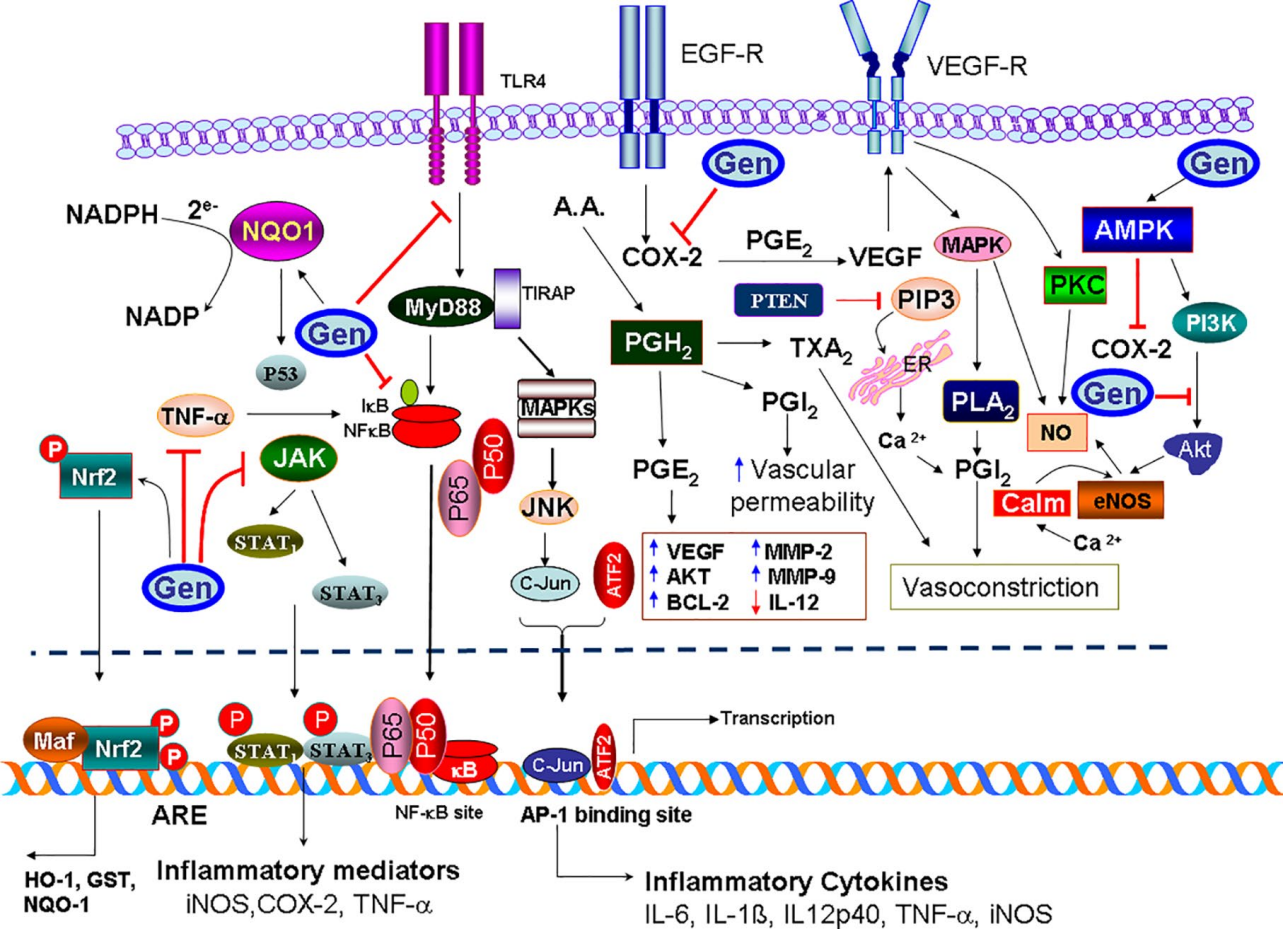
Illustration of antimetastatic potential of genistein (Gen) in cancer. Genistein activates genistein-mediated activated protein kinase (AMPK)/mitogen-activated protein kinase (MAPK)/protein kinase C (PKC) pathways; the downstream of these sequences is the activation of neuronal nitric oxide synthase (nNOS), which activates the production of NO that possess a strong antioxidant/anti-inflammatory activity. Genistein also inhibits the activity of cycloxygenase-2 (COX-2), whether through the activation of AMPK pathway, or by inactivation of PIP3 pathway. Notably, COX-2 causes upregulation of vascular endothelial growth factor (VEGF) and VEGF-R activation. Further, the overexpression of COX-2 promotes the production of PGH_2_, which further metabolized to *prostaglandin E_2_* *(PGE_2_)*, prostacyclin (PGI2), and thromboxane A_2_ (TXA_2_). Genistein inactivates TLR4 dimerization, thus abolishes MyD88 or TIR-domain-containing adapter-inducing interferon-β (TRIF) to activate nuclear factor-κB (NF-κB), so inhibit its translocation into the nucleus, and that prevents the upregulation of proinflammatory cytokines. Genistein possesses a strong inhibitory effect on oxidative stress by activating the nuclear factor erythroid 2-related factor 2 (Nrf2)-heme oxygenase-1 (HO-1)/NAD(P)H dehydrogenase (quinone) 1 (NQO1) pathway. Since, genistein is able to activate the Nrf2/NQO1 pathway. After being phosphorylated, Nrf2 activates antioxidant response element (ARE) and enhances transcription of Nrf2-regulated genes including NQO-1, HO-1, and glutathione S-transferase (GST). Also, Genistein has a potent anti-inflammatory effect through prevention of tumor necrosis factor-α (TNF-α)-induced NF-κB translocation into the nucleus, and inhibition of MAPKs/c-Jun N-terminal kinase (JNK)/c-Jun pathway, which decline the induction of the inflammatory mediators. Genistein also abolishes the activation of signal transducer and activator of transcription 1 (STAT1)/signal transducer and activator of transcription 3 (STAT3) and prevents their translocation into the nucleus through downregulating the activity of JNK.

The cancer metastasis is one of the most prominent causes of death worldwide. The metastasis of cancer is found to be dependent on higher expression of MMPs (**Figure 7**). In a study using nude mice, genistein was found to inhibit the metastasis of salivary adenoid cystic carcinoma cells (ACC) by inhibiting the VEGF and MMP-9 expression (Liu and Yu, 2004). Further evidences suggested that genistein displayed an inhibitory effect on the migration of MAT-LyLu and AT-2 rat prostate cancer cells (Miekus and Madeja, 2007).

The expression and phosphorylation of focal adhesion kinase (FAK) in hepatocellular carcinoma cells (MHCC-97H) were found to be decreased. Furthermore, *in vivo* xenograft transplantation of MHCC-97H cells have shown that number of pulmonary micrometastatic foci was significantly lowered in comparison to control group (Gu et al., 2009). Studies using colon cancer cells (HCT116) revealed that genistein-mediated antimetastatic effects *via* inhibition of COX-2 and MMP-9, Ang-1, vasodilator-stimulated phosphoprotein (VASP) and VEGF (Kang et al., 2018). Other investigators have reported the downregulation of ERK1/2, PI3K/Akt and MMP-2 in genistein-treated lung cancer cells (A549) (Noori-Daloii, 2012). Similarly, the downregulation of phosphorylated focal adhesion kinase, p-paxillin, tensin-2, vinculin, and α-actinin has been observed in genistein-treated melanoma cells (B16F10). Furthermore, the expression of Snail, an epithelial-to-mesenchymal transition transcription factor was found to be decreased following genistein treatment (Cui et al., 2017).

Unlike specific chemotherapeutic drugs, which target specific pathway, the mechanism of genistein is wide and involve a spectrum of molecular pathways. All these effects of genistein could increase its antimetastatic effect and deter the invasion and spread of tumor cells into other tissues which, at least, may alleviate the pain and suffering of cancer patients.

### Anti-Inflammatory and Antioxidant Effects

It is well understood that inflammatory behavior is closely related to cancer development. Cancer cells express a variety of inflammatory mediators, such as cytokines and chemokines. Literature suggested that anticancer drugs can be used to treat inflammatory responses. Previous studies have reported the inhibitory effect of genistein on signal transducer and activator of transcription 1 and NF-κB-mediated signaling cascade in activated macrophages (Hamalainen et al., 2007). Other researchers reported that genistein suppressed inflammatory responses through inhibition of COX‐2, phosphorylated c-Jun N-terminal kinase (JNK), protein kinase R-like ER kinase, and pp38 and *in vitro* and *in vivo* studies MCF-7 breast cancer cell line and mammary glands of female rats, respectively, (Hwang et al., 2009). Studies using RAW 264.7 macrophages and MH7A human synoviocyte cells have also reported the downregulation of inflammatory mediators, such as IL-1β, IL-6, IL-8, TNF-α, IL-6, and NF-κB (Ji et al., 2012; Li et al., 2014). In addition, genistein was found to attenuate proinflammatory responses in lipopolysaccharide-induced microglia cells (BV2). Genistein repressed the production of NO and prostaglandin E_2_ by downregulating the expression of nitric oxide synthase and COX-2 in microglial cells. In addition, the expression of inflammatory cytokines, including IL-1β and TNF-α, were also markedly suppressed by genistein through inactivation of NF-κB and toll like receptor-4 (Jeong et al., 2014). The administration of genistein (1.04 or 1.3 mg/day) was found to lower the expression of TNF-α and IL-6 in a murine model of peritoneal endometriosis, indicating its ability to inhibit the development of inflammation (Sutrisno et al., 2018). More recently, researchers have reported the anti-inflammatory and anticancer response of long-term genistein treatment in diethylnitrosamine-induced hepatocellular carcinoma in mice (Lee et al., 2019). In conclusion, it’s very clear that the possible clinical application of genistein as an anti-inflammatory agent could be due to its ability to block many inflammatory cascades that help cancer cells to create their own and independent microenvironment. The inhibition or attenuation of the inflammatory mediators, which encompass growth factors, chemokines, and cytokines, could suppress the function of cancer cells.

In other context, free radicals are constantly produced during oxidative stress, and they are known to be highly toxic and have a deleterious effect on the cellular and enzymatic integrity. Genistein plays an important role on recycling enzymes, which are known as body’s main antioxidant constituents. For instance, genistein induced activation of antioxidant enzymes through AMPK and PTEN pathways in prostate cancer cells (DU145). Genistein also stimulated the expression of the manganese superoxide dismutase and catalase, which collectively led to significant reduction in reactive oxygen species levels (Park et al., 2010). *In vivo*, genistein exposure governed the non-enzymatic antioxidant action by increasing the expression of glutathione and NAD(P)H dehydrogenase (Wiegand et al., 2009; Vanhees et al., 2013). In another study, researchers revealed that genistein possessed inhibitory action on cancer-associated enzyme, ornithine decarboxylase, and considerably suppressed skin carcinogenesis stimulated by ultraviolet light in a mice model (Record et al., 1995; Mazumder and Hongsprabhas, 2016).

As described above, there is a complex interference between the inflammatory mediators and oxidative stress. Collectively or separately, each potentiate the production of the other, and both worsen the status of cancer patients. As discussed above, genistein has the potency to attenuate the inflammatory mediators aside its ability to increase the activity of many antioxidant enzymes. That could be helpful in battle against cancer to improve the lifestyle of cancer patients and relieve their pain due to such inflammation. Nevertheless, further clinical studies are required to investigate the therapeutic potential of genistein in cancer patients.

### Genistein and miRNA

The microRNAs (miRNAs) are a class of small noncoding RNAs, consisting of 19–24 nucleotides, which regulate almost 60% of genes in the human genome (Macfarlane and Murphy, 2010). Emerging pieces of evidence have suggested that miRNAs are deregulated in almost all human cancers, and found to involve in a wide spectrum of cancer hallmarks, such as sustained proliferative signaling, evasion of growth suppressors, activated invasion and metastasis, replicative immortality, angiogenesis, resistance to cell death, and evasion of immune destruction (Van Roosbroeck and Calin, 2017). Based on previous studies, miRNAs have also been widely proposed as potential targets for treating various cancers. So, it is very important to know how genistein influences the expression and regulation of miRNAs in human cancer cells.

Recently, Lynch and colleague (2016) have demonstrated that following treatment with 40 µM genistein for 7 days in PC3 prostate cancer cell line, the expression of miR-200c significantly increased, while the methylation levels of miR‐200c gene get reduced (Lynch et al., 2016). Genistein can affect and regulate the expression of noncoding RNAs, especially microRNAs (miRNAs), in cancer cells (**Table 2**).

**Table 2:** Effect of genistein on cancer-associated microRNAs (miRNAs).

Cancer	Cell lines	Duration of treatment with concentration	Effect on miRNA expression with fold change	Gene/Proteins/Pathways affected	References
Prostate cancer	PC3	7 days (40 μM)	↑miR‐200c	ZEB1	Lynch et al. (2016)
				ZEB2	Mongroo and Rustgi (2010)
				Slug	Liu et al. (2013)
				EMT	Feng et al. (2014)
	PC3, DU145	4 days (25 μM)	↓miR-1260b (∼2-fold);↓miR-151;↑miR-574-3p	sRRP1 Smad4	Chiyomaru et al. (2012); Chiyomaru et al. (2013); (Hirata et al., 2014)
Breast cancer	MDA-MB-435	48 h (5 μM)	↓miR-155 (∼ 1.8-fold)	Forkhead box 03, PTEN, casein kinase and p27	De la Parra et al. (2016)
Human Laryngeal cancer			↑miR-1469	Mcl1	Ma et al. (2018)
Multiple myeloma	U266	2 days (40 μM)	↑miR-29b (∼2-fold)	NF-κB	Xie et al. (2016)
Pancreatic cancer			↓miR-223	F-box/WD repeat-containing protein 7 gene	(Ma et al., 2013)

The miR‐200 family was found to act as a tumor‐suppressive group of miRNAs that targets *ZEB1*, *ZEB2*, and *Slug* genes, which drive epithelial–mesenchymal-transition and tumor progression in prostate cancer cells (Mongroo and Rustgi, 2010; Liu et al., 2013; Feng et al., 2014). Similarly, in another study, it was demonstrated that after treatment with 25 µM genistein for 4 days in PC3 and DU145 prostate cancer cell lines, the level of miR-1260b expression was significantly decreased (∼2-fold) compared to nongenistein treated cells (Hirata et al., 2014). The miRNA-1260b expression in human prostate cancer cell lines was found to be significantly higher in prostate cancer tissue and cells with concomitant increase in cell proliferation, migration, and invasive capacity of prostate cancer cells (Hirata et al., 2014).

Mechanistically, genistein exerted its antitumor effect *via* downregulation of miR-1260b that targets sRRP1 and Smad4 genes *via* DNA methylation or histone modifications, at least in part in prostate cancer cells (Hirata et al., 2014). Similarly, in another study, it was shown that treatment of PC3 and DU145 cells with 25 µM genistein downregulated the expression of miR-151. Inhibition of miR-151 in prostate cancer cells by genistein significantly suppressed cell migration and invasion (Chiyomaru et al., 2012). It was also found that genistein upregulated the tumor suppressor miR-574-3p expression and significantly affected cell proliferation, migration, and invasion in prostate cancer cell lines (Chiyomaru et al., 2013) In the case of breast cancer, 5 µM of genistein significantly reduced (∼1.8-fold) the oncogenic miR-155 expression in MDA-MB-435 cells (De la Parra et al., 2016). In parallel with the reduction of miR-155, the levels of proapoptotic and antiproliferative proteins, such as forkhead box 03, PTEN, casein kinase, and p27, also get upregulated in MDA-MB-435 cells in response to genistein treatment. In the case of laryngeal cancer, it has shown that genistein induced the expression of miR-1469, which, in turn, promoted cell apoptosis and inhibited the expression of myeloid cell leukemia 1, an antiapoptotic member of the Bcl-2 family of apoptosis-regulating proteins, thus preventing human laryngeal cancer (Ma et al., 2018). In the case of multiple myeloma cells, it was observed that following treatment with genistein (40 µM) for 48 h, the expression of miR-29b in U266 multiple myeloma cells was significantly increased (two-fold) (Xie et al., 2016). It was further demonstrated that miR-29b significantly inhibited NF-κB expression and increased apoptosis in U266 multiple myeloma cells (Xie et al., 2016). In pancreatic cancer cells, genistein treatment significantly inhibited miR-223 expression and upregulated F-box/WD repeat-containing protein 7 gene, one of the targets of miR-223 (Ma et al., 2013). Through the above studies, one can conclude that genistein induces or reduces the expression of miRNAs in cancer cells. Nevertheless, further investigations are still required to unravel the precise mechanism of action of genistein on miRNAs regulation.

### Combinatorial and Clinical Studies Using Genistein

Genistein exhibited synergistic apoptotic and anti-inflammatory effects with capsaicin in MCF-7 human breast cancer cells by modulating AMPK and COX-2 (Hwang et al., 2009). In HepG2 human hepatocellular carcinoma cells, the extent of apoptosis was maximal when genistein exposure for 24 h was followed by oestradiol treatment for 48 h as compared to either of the agents alone (Sanaei et al., 2017). Moreover, genistein potentiated the anticancer action of chemotherapeutic drug 5-fluorouracil in MIA PaCa-2 human pancreatic cancer cells by increasing both apoptotic as well as autophagic cell death. Studies in animals transplanted with MIA PaCa-2 cells also confirmed these observations, revealing a significant decrease in tumour volume with combined treatment regimen (Suzuki et al., 2014). In addition, efficacy of photofrin-mediated photodynamic therapy to induce apoptosis was considerably enhanced by genistein against SK-OV-3 human ovarian cancer and SNU 80 human anaplastic thyroid cancer cells (Ahn et al., 2014). These effects were regulated by activation of the general apoptotic signaling cascade requiring activation of caspase-8 and caspase-3 (Ahn et al., 2012; Ahn et al., 2014).

In view of chemopreventive potential of genistein based on *in vitro* and *in vivo* studies, genistein research has been upscaled to study its safety and efficacy in clinical trials either individually or in combination with other chemotherapeutic agents. Although there are only few clinical trials that have attempted to study the safety and efficacy of genistein, these studies have presented promising results which are being summarized in this section. In a 12-month, randomized, placebo-controlled study, Atteritano et al. (2008) investigated the effect of genistein at a dose of 54 mg/day on cytogenetic biomarkers in peripheral lymphocytes of postmenopausal women (*n* = 57) and reported that the administration of genistein effectively reduced cytogenetic markers. Following 1-year of genistein administration, sister chromatid exchange rate was 3.98 ± 1.14 (p < 0.05), high frequency cells count dipped to 3% from initial count of 5% and chromosomal aberration frequency was documented to be 4.5% (p < 0.05), emphasizing the positive effect on reduction of cytogenetic biomarkers. In another randomized, double-blind, placebo controlled study, the administration of genistein in postmenopausal women with type 2 diabetes mellitus (*n* = 28) was reported to improve fasting blood glucose, serum triglyceride, glycated hemoglobin, and malondialdehyde levels along with control of oxidative stress levels (Braxas et al., 2019). Similar results were reported from another study where use of genistein in a randomized, double blind, placebo-controlled clinical trial was reported to be safe for postmenopausal women (Delmanto et al., 2013). In another phase I/II pilot study (*n* = 13), genistein in combination with FOLFOX/FOLFOX-bevacizumab was found to be safe and torable for metastatic colorectal cancer patients with 61.5% overall response rate and 11.5 months of median progression free survival (Pintova et al., 2019). Schneider et al. (2019) reported safety profile of administration of PhytoSerum (containing genistein, daidzein, and S-equol) in a phase 1/2a randomized clinical trial, where the formulation at daily dose of 50 or 100 mg was well tolerated by perimenopausal women. In a phase I dose escalation study in pancreatic cancer patients (*n* = 16), administration of AXP107-11 (crystalline form of genistein) along with gemcitabine illustrated favorable pharmacokinetic profile with mean overall survival of 4.9 months (Lohr et al., 2016). In presurgical bladder cancer patients (phase 2 trial of genistein), genistein was shown to be more effective at a lower dose to reduce EGFR phosphorylation in bladder cancer tissue (Messing et al., 2012). The safety and efficacy of genistein was further documented from another double-blind phase 2 clinical trial in localized prostate cancer patients (Lazarevic et al., 2011). These studies depict the chemopreventive nature of genistein and its safety and efficacy alone or in combination with other chemotherapeutic agents. These initial results from clinical studies further strengthen the potential of genistein as a chemopreventive drug and encourages further studies.

## Current Limitation and Challenges

Despite knowledge gained and discussed above and the tremendous clinical success in cancer therapy in the present era, the clinical application of genistein as a promising therapeutic agent for cancer treatment is not fully understood. However, one of the major limitations of currently available anticancer drugs is the ability of cancer cells to develop chemoresistance against such drugs. Cancer cells are able to produce multidrug resistance proteins, such as multidrug resistance protein 1, and multidrug resistance-associated protein 1 (ABCC1), which causes drug resistance. Therefore, chemoresistance represents a serious clinical obstacle in effectively treating cancer patients. Polo-like kinase 1 (Plk1) has been shown to be involved in chemoresistance, so Plk1-targeted therapies could possibly reduce or eliminate the chemoresistance of cancer cells against anticancer drugs. Genistein was proposed as a Plk1 inhibitor, which effectively downregulates the expression of multidrug resistance protein 1 and multidrug resistance-associated protein 1, key factors inducing chemoresistance in paclitaxel-resistant cancer cells (Shin et al., 2019). The discovery of genistein being a Plk1 inhibitor adds a new horizon for the possible application of genistein as adjuvant in chemotherapy because of its ability to prevent cancer cells from acquiring resistance against the anticancer drugs or due to resensitize drug-resistant cancer cells.

The other limitation in our knowledge is that as a natural compound genistein may represent a raw material we can further work on, to improve its characters, which may improve the quality of lifestyle of cancer patients. For instance, in a murine xenograft study using human prostate cancer cell lines, the synthetic genistein nanosuspension (BIO 300) improves the therapeutic index in prostate cancer treatment by preventing radiation-induced erectile dysfunction without reducing tumor radiosensitivity (Jackson et al., 2019). In fact the low aqueous solubility of genistein is the major problem that limits the clinical development and applications of genistein, thus modification of its chemical structure can improve its aqueous solubility and contribute to higher bioavailability (Tang et al., 2019). Additionally, the stability of genistein is another critical factor which affects its bioavailability. Methylation of the free hydroxymethyl group of genistein enhances its metabolic stability and improves the membrane transportation capacity (Chiang et al., 2017). The question is that as one of the main dietary source of genistein, can we increase the health benefits of soybean. Based on the available knowledge, yet that question has not been answered. However, a recent study may partially give us an avenue for this purpose. Treatment of soybeans with ultrasound increased its isoflavone contents and improved the functional properties related to their health benefits (e.g. increased bioactivity as radical scavengers and by reducing the activation of NF-κB (Falcao et al., 2019). As discussed above, the clinical importance of NF-κB activation is derived mainly from its role in inflammatory responses and apoptosis cascades. Fermentation also has been suggested as a possible way to improve the bioavailability of genistein and further accelerates its absorption (Lee et al., 2017). But again one can ask the question whether such increase in the bioavailability of isoflavones in soybeans is safe. That is in fact dependent on the level of dietary intake of isoflavones. In a randomized double-blind parallel study in men with type-2 diabetes mellitus, and in a double-blind randomized parallel study on bone turnover markers in women within 2 years of onset of menopause, the high-dose isoflavone intake impaired thyroid function (Sathyapalan et al., 2017a; Sathyapalan et al., 2017b). There are also inherent challenges in the effectiveness of genistein on different human cohorts. In a study monitoring the short-term versus long-term effect of genistein treatment in prostate cancer, short term treatment of PC3-M cells (metastatic prostate cancer cells) *in vitro* over a period of 8 weeks decreased MMP-2 expression whereas long-term treatment up to 2 months increased MMP-2 levels, emphasizing the fact that early phase chemoprevention trails need to be closely monitored for optimal dose administration duration for maximum efficacy (Zhang et al., 2019). These data indicate that biomarker expression can change as a function of treatment time. Therefore, there is still an urgent need for performing further studies to optimize the effective doses and the best route of administration for genistein, whether in cancer therapy and other types of illnesses. Further, genistein is known as a strong antioxidant compound and can provide protection against oxidative stress in various noncancerous tissues. For instance, treatment with genistein was shown to preserve follicular quality in rats with polycystic ovary syndrome by elevating antioxidant capacity (Rajael et al., 2019), increased the total antioxidant activities and reduced kidney damages in nephrotic rats (Javanbakht et al., 2014), or improved antioxidant status and decreased pancreatic damage following administration of nicotine to mice (Salahshoor et al., 2019). In concert with its multitargeted anticancer activities, protective action of genistein on adjacent normal healthy tissues makes this natural isoflavone an attractive key compound for development of novel and more effective drug for combating malignant disorders in the future.

## Conclusions and Future Perspectives

The present manuscript extensively reviewed the available scientific literature on the potential role of genistein as an anticancer agent with detailed description of its targets in signaling cascades. The *in vivo* and *in vitro* studies further emphasized the chemopreventive potential of genistein, in view of which genistein has been upscaled to clinical trials. This is mainly promising for the patients in developing countries where currently marketed therapies for cancer treatment are very expensive and beyond the reach of patients, thereby limiting the affordability of treatment options. Thus, developing countries are desperately looking for low cost options for cancer treatment and prevention, of which natural products as anticancer agents seem promising. This further emphasizes the need to develop safe and effective natural product-derived agents as chemopreventive drugs which are easily available. Genistein (an isoflavone) is one among bioactive natural molecules with aromatic substitution at carbon C3 instead of the C2 position as it is common for other types of flavonoids. Considering the involvement of genistein in a wide-variety of molecular pathways, there is a greater need to understand this key molecule in terms of its regulatory role as well as its therapeutic potential. The clinical and experimental evidences gathered in this review are suggestive of the promising therapeutic role of genistein in the treatment and prevention of different types of cancer and encourage further *in vivo* studies with this attractive natural molecule. More recently, (Shaji, 2018) investigated the interactions of genistein with monocarboxylate transporter-8 to treat Allan–Herndon–Dudley syndrome. A very recent study has shown the promising role of genistein in combination with chemotherapeutic drug FOLFOX to treat metastatic colorectal cancer (Pintova et al., 2019). Therefore synergistic approaches using genistein may help in near future to treat grave diseases, such as cancer.

Although we have a come a long way ahead in cancer treatment modalities, we still have unexplored dimensions to scale for modulating effective treatment strategies for cancer patients with long-term safety and efficacy. Natural products, such as genistein, have been extensively studied for its anticancer effects which are summarized in this review. However, we still have a long way to go. First, besides the known molecular targets as therapeutic modulators, further clinical studies on large patient cohorts are required to extensively study the chemopreventive and therapeutic potential of genistein in conjunction with its long-term safety and efficacy in treating cancer patients. The drug–drug interactions of genistein with other chemotherapeutic agents need to extensively studies on both experimental and clinical front to maximize the efficacy and minimize drug resistance and risk of replase. Secondly, the possible mechanism and strategies for increasing the accessibility and bioavailability of genistein need to be explored in more details. Thirdly, the precise therapeutic dose of genistein for treatment of different cancer types needs to be standardized. Fourthly, the duration and time of genistein dose administration need extensive standardization based on variable effects reported in long-term and short-term treatment administration regimens. Furthermore, the role of nanotechnology should also be explored in future to reduce the required dosses of genistein and to specifically target tumor tissues using novel drug delivery techniques. Recently, using encapsulated genistein nanomaterials, Pool et al. (2018) have investigated the promising anticancer potential of genistein in HT29 human colon cancer cells *via* activation of apoptosis and autophagy along with H_2_O_2_ production. In addition, the utilization of molecular docking studies to uncover the hidden mechanisms of action of genistein should also be explored. Hence, extensive preclinical research along with clinical studies needs to be designed and executed before genistein is incorporated as one of the mainstream treatment of choices for cancer. In view of available literature, genistein presents as a promising chemopreventive drug specially with respect to management of drug resistance in cancer patients which is of clinical interest. Nevertheless, extensive work needs to be done to translate the therapeutic efficacy of genistein from bench to bedside.

## Author Contributions

HT performed literature survey and data extraction. MT contributed sections on apoptosis, cell cycle arrest, antiangiogenesis, anti-inflammatory and conclusion and also created figures of the indicated sections. FT participated in data extraction. KS extracted data and wrote the abstract and introduction section. MK prepared the section on the chemistry of genistein. AS and VA proofread the manuscript. US and AJ composed the text on miRNA and genistein. VA and HT revised the manuscript. AB critically reviewed and edited the manuscript and also made suggestions for improvements. All authors read and approved the final manuscript.

## Conflict of Interest

KS is recruited by the company NGO Praeventio, Tartu, Estonia.

The remaining authors declare that the research was conducted in the absence of any commercial or financial relationships that could be construed as a potential conflict of interest.
